# Deferoxamine Suppresses Collagen Cleavage and Protease, Cytokine, and COL10A1 Expression and Upregulates AMPK and Krebs Cycle Genes in Human Osteoarthritic Cartilage

**DOI:** 10.1155/2016/6432867

**Published:** 2016-11-30

**Authors:** Elena V. Tchetina, Galina A. Markova, A. Robin Poole, David J. Zukor, John Antoniou, Sergey A. Makarov, Aleksandr N. Kuzin

**Affiliations:** ^1^Immunology and Molecular Biology Laboratory, Nasonova Research Institute of Rheumatology, Moscow, Russia; ^2^Department of Surgery, McGill University, Montreal, QC, Canada; ^3^Jewish General Hospital, McGill University, Montreal, QC, Canada; ^4^Surgery Department, Nasonova Research Institute of Rheumatology, Moscow, Russia; ^5^Forensic Medicine Service, Moscow City Health Department, Moscow, Russia

## Abstract

This study reports the effects of the iron chelator deferoxamine (DFO) on collagen cleavage, inflammation, and chondrocyte hypertrophy in relation to energy metabolism-related gene expression in osteoarthritic (OA) articular cartilage. Full-depth explants of human OA knee articular cartilage from arthroplasty were cultured with exogenous DFO (1–50 *μ*M). Type II collagen cleavage and phospho-adenosine monophosphate-activated protein kinase (pAMPK) concentrations were measured using ELISAs. Gene expression studies employed real-time PCR and included AMPK analyses in PBMCs. In OA explants collagen cleavage was frequently downregulated by 10–50 *μ*M DFO. PCR analysis of 7 OA patient cartilages revealed that 10 *μ*M DFO suppressed expression of MMP-1, MMP-13, IL-1*β*, and TNF*α* and a marker of chondrocyte hypertrophy, COL10A1. No changes were observed in the expression of glycolysis-related genes. In contrast, expressions of genes associated with the mitochondrial Krebs cycle (TCA), AMPK, HIF1*α*, and COL2A1 were upregulated. AMPK gene expression was reduced in OA cartilage and increased in PBMCs from the same patients compared to healthy controls. Our studies demonstrate that DFO is capable of suppressing excessive collagenase-mediated type II collagen cleavage in OA cartilage and reversing phenotypic changes. The concomitant upregulation of proanabolic TCA-related gene expressions points to a potential for availability of energy generating substrates required for matrix repair by end-stage OA chondrocytes. This might normally be prevented by high whole-body energy requirements indicated by elevated AMPK expression in PBMCs of OA patients.

## 1. Introduction

Osteoarthritis (OA) is a systemic condition that may affect single or multiple joints. OA involves degenerative changes in articular cartilage, remodelling of subchondral bone, and limited synovial inflammation [1]. Osteoarthritic changes in articular cartilage are associated with progressive proteolytic degradation of the extracellular matrix (ECM), which is composed mainly of type II collagen and aggrecan. Their destruction leads to eventual cartilage loss that is accompanied by phenotypic hypertrophy-related changes in chondrocytes, such as the production of type X collagen (a marker of hypertrophy) and upregulation of collagenase MMP-13, events similar to those seen in the foetal growth plate [1–3].

It is generally accepted that net collagen loss in the ECM in OA articular cartilage results from net collagen degradation, which outweighs any increase in collagen synthesis [1]. Protein synthesis is a process that consumes substantial energy and, thus, requires sufficient amounts of ATP [4]. Cellular ATP concentrations are controlled by adenosine monophosphate-activated protein kinase (AMPK), the expression of which is regulated by the AMP/ATP ratio [5, 6]. In addition, protein synthesis requires amino acids, which are produced by transamination of intermediates of the glycolytic pathway, the pentose phosphate pathway, or the Krebs cycle (citric acid, tricarboxylic acid, TCA cycle) [7].

Recently, mitochondrial dysfunction, accompanied by a decrease in AMPK expression in OA chondrocytes and in healthy articular chondrocytes in the presence of interleukin- (IL-) 1*β* or tumor necrosis factor (TNF)*α*, has been reported [8, 9]. Moreover, AMPK activators can reverse chondrocyte procatabolic responses [9]. Limitations in the required amino acids may, therefore, reduce OA chondrocytes capacity of producing sufficient amounts of collagen even under conditions of high ATP availability.

AMPK expression [10, 11] and type II collagen synthesis [12, 13] are increased in healthy chondrocytes under hypoxic conditions. Low oxygen tension stimulates hypoxia-inducible factor (HIF) expression in normal and OA chondrocytes* in vitro* that accompanies increased expression of ECM-related genes and causes decreased expression of degradation markers and chondrocyte hypertrophy [14].

HIF1*α* induction under normoxic conditions by the hypoxia-mimicking agent and iron chelator o-phenanthroline also reduces MMP-13 and MMP-3 expression but has limited ability to improve matrix synthesis during chondrogenic differentiation in human osteoarthritic articular chondrocytes [15, 16]: this might be associated with concomitant inhibition of mitochondrial respiration [17].

In contrast, another iron chelator deferoxamine (DFO) is capable of stabilizing HIF1*α* expression while simultaneously improving dental pulp cell repair activity [18] and accelerating wound healing in diabetic rats [19]. The effects of DFO are associated with the upregulation of glycolysis in cancer cells under normoxic conditions [20, 21], and the preservation of respiratory chain cytochrome-c-oxidase subunit concentrations, which might enhance aerobic respiration during hypoxia [22, 23].

Based on these observations, we hypothesized that DFO treatment of human OA articular cartilage explants might decrease collagen cleavage activity and generation of proinflammatory cytokines and recover an often-altered chondrocyte phenotype by changing the cellular energy balance in favour of improving of mitochondrial function and restoring an anabolic matrix phenotype. In this study, we test this hypothesis in cultured OA cartilage while also examining and comparing whole-body AMPK expression by peripheral blood mononuclear cells (PBMCs) in OA versus normal patients.

## 2. Patients and Methods

### 2.1. Ethics

The study was in compliance with the Helsinki Declaration. The study protocol was approved by the McGill University and the Nasonova Research Institute of Rheumatology (Protocol number 5, February 17, 2011) Committees on the Ethics of Human Research, and informed consent was obtained from all subjects prior to performing the study.

### 2.2. Patients

Human femoral condylar cartilage samples were obtained via total knee arthroplasty from 44 OA patients (21 men, mean age 66.2 ± 11.2 years, range 53–79 years; 23 women, mean age 64.4 ± 12.4 years, range 46–90 years): 7 patients were used for DFO dose-response studies; 15 patients, to study the inhibition of collagenase cleavage activity caused by DFO; and 7 patients, to study the effects of DFO on gene expression. Knee articular cartilages from another 15 OA patients from whom peripheral blood was also obtained were used to examine AMPK gene expression. Condylar cartilages from 2 OA patients were used for DFO toxicity studies. As articular cartilage was obtained after joint replacement surgery, the amount of tissue was limited and scarcely sufficient for one assay procedure. Therefore, different assays required the use of different patients. All end-stage OA arthroplasty patients had radiological K&L grades of III or IV, experienced severe pain, and had walking problems (lameness). All of the patients studied fulfilled the OA criteria of the American College of Rheumatology [24].

Human articular cartilage from 12 healthy individuals (10 men, mean age 36.0 ± 7.7 years, range 25–45 years; 2 women, mean age 35.0 ± 2.1 years, range 34–37 years) was obtained less than 12 h postmortem at autopsy from the femoral condylar surfaces of the knee that articulate with the patella. No patient had received chemotherapy, was diagnosed with diabetes, or exhibited lower limb vascular insufficiency.

We also examined PBMCs from 15 end-stage OA patients, aged 49 to 71 years (mean age 56.6 ± 8.9 years) undergoing knee joint arthroplasty. For the control group, peripheral blood was also obtained from 16 healthy subjects (average age 58.6 ± 8.3 years, range 42–74 years) who were free of any serious diseases.

### 2.3. Cartilage Isolation and Preparation

Femoral condylar articular cartilage samples were isolated and prepared for culture as previously described [25]. All OA cartilage samples exhibited macroscopic articular surface differences compared with normal cartilages. To generate sufficient cartilage to perform each patient analysis, all the available articular cartilage from each patient, regardless of the degree of degeneration (Mankin grades 4–12), was used as we have previously described [25]. For Mankin grading of OA articular cartilage the specimens were removed as full-depth (to subchondral bone) slices cut from three different pieces from the same knee. Each slice was graded; the mean value was considered as an averaged value. Articular cartilage specimens with normal cartilage (as revealed by Mankin grade of 1) from donors were removed at autopsy as full-depth (to subchondral bone) slices cut from the femoral condyle surface that articulates with patella: from medial (2 pieces) and lateral (2 pieces) condyles and from the patella-femoral groove (3 pieces) as shown on Supplementary Figure (see Supplementary Material available online at http://dx.doi.org/10.1155/2016/6432867). Cartilage pieces from all the sites of one knee were combined before RNA isolation.

The OA articular cartilage was washed three times with high glucose Dulbecco's modified Eagle's medium A (DMEM; Gibco BRL, Life Technologies, Grand Island, NY, USA) containing 20 mM HEPES buffer (pH 7.4) (Gibco BRL), 45 mM NaHCO_3_, 100 units/mL penicillin, 100 *μ*g/mL streptomycin, and 150 *μ*g/mL gentamycin sulfate. Full-depth cartilage slices of approximately 20 mm × 20 mm were cut vertical to the articular surface and then into cubes. Five to seven cubes were randomly obtained and wet weights of approximately 50 mg were distributed into each culture well (48-well Costar 3548 plate; Corning Inc., Corning, NY). Prior to DFO addition, samples were maintained for 48 h at 37°C in 1 mL per well of medium A in 95% air and 5% CO_2_.

### 2.4. Cartilage Explant Culture

Media were changed after 48 h (day 0). OA articular explants cultured without DFO were used as a control. Final concentrations of 1–50 *μ*M DFO (Sigma) were freshly added to medium A from day 0 at each medium change every 4 days. Cartilage (triplicate cultures for each analytical point) was cultured for a total of 16 days and analyzed at day 16 for type II collagen cleavage by collagenases. The conditioned media were collected every 4 days at each medium change from days 4 to 16 and stored at −20°C until analyzed [26]. For gene expression analysis, separate cultures were maintained for 48 h and analyzed as described below.

### 2.5. Toxicity Assays

Toxicity was measured as described previously [27]. In brief, after the media change at day 0, DFO was added in concentrations from 1–50 *μ*M to the OA articular cartilage explants and cultured for 4 days. After the media change, the cartilage was biolabeled by adding 25 *μ*Ci/mL of tritiated proline or 10 *μ*M/mL of tritiated thymidine (Amersham, Oakville, Ontario, Canada). The cartilage was cultured for 4 more days. The cartilage was then washed 3 times for 1 h each in phosphate buffered saline (PBS) to remove any free label. The cartilage was digested overnight with 1 mL proteinase K (Sigma) at 1 mg/mL in 50 mM Tris HCl, pH 7.6. The digest was mixed with a scintillation fluid (Ready Value; Beckman Instruments, Irvine, CA, USA) and analyzed with a Packard CA 1900 scintillation counter (Meriden, CT, USA).

### 2.6. ELISA Assays of Collagenase-Cleaved Type II Collagen

OA cartilage explants from day 16 of culture were digested and extracted with *α*-chymotrypsin to solubilize the denatured collagen, including the carboxyterminal neoepitope COL2-3/4C short (C1, 2C) epitope generated by the cleavage of type II collagen by collagenase: extracts and media were assayed as described previously by ELISA [25]. Total cleavage neoepitope in the cartilages and media was calculated using a summation of the data from each medium change and cartilage analysis. The results are expressed as pmoles of the epitope/mg wet weight of cartilage, which is based on a molecular weight of the standard peptide epitope of 608 g/mol. Collagen cleavage was analyzed separately in each OA patient and mean values were recorded.

### 2.7. Peripheral Blood Fractionation

Peripheral blood (10 mL) was collected in Vacutainer tubes containing ethylenediaminetetraacetic acid (EDTA) (BDH, England). Blood samples were taken in a standardized manner in the morning (between 07:00 AM and 09:00 AM). Whole blood fractionation was performed using a Ficoll density gradient [28]. PBMCs were collected, washed twice in phosphate buffered saline (PBS), and frozen at −70°C prior to protein extraction.

### 2.8. Quantification of pAMPK Protein Levels

Concentrations of phospho-AMPK (cat. number KHO0651) were determined in isolated PBMCs lysates by ELISA (Invitrogen, Camarillo, CA, USA) according to the manufacturer's instructions. Lysates were prepared using a cell extraction buffer containing 10 mM Tris, pH 7.4, 100 mM NaCl, 1 mM EDTA, 1 mM EGTA, 1 mM NaF, 20 mM Na_4_P_2_O_7_, 20 mM Na_3_VO_4_, 1% Triton X-100, 10% glycerol, 0.1% SDS, and 0.5% deoxycholate (Invitrogen, Camarillo, CA, USA) supplemented with protease inhibitor cocktail (Sigma-Aldrich, Inc., St. Louis, USA) and 1 mM PMSF (Sigma-Aldrich, Inc., St. Louis, USA) according to the manufacturer's instructions. Total DNA content in PBMC lysates was measured spectrophotometrically using GeneQuant (Amersham Biosciences, United States). Results were expressed per *μ*g of DNA.

### 2.9. Total RNA Isolation and Reverse Transcription (RT)

For detection of gene expression, total RNA was isolated from fresh knee articular cartilage and PBMCs using TRIzol reagent according to the manufacturer's recommendations (Invitrogen, Carlsbad, CA, USA). Total RNA had an *A*_260/290_ > 1.9. The RT-reaction was performed using a Reverta kit containing M-MLV Reverse Transcriptase, random hexanucleotide primers, and total RNA according to the manufacturer's recommendations (InterLabService, Moscow, Russia).

### 2.10. Real-Time Quantitative PCR

Premade primers and probes for TaqMan assay (Applied Biosystems, Foster City, CA, USA) of human genes were MMP1 (Hs00233958_m1); MMP-13 (Hs00233992_m1); CTSК (Hs00166156_m1); COL10A1 (Hs00166657_m1); Glut1 (Hs00197884_m1); PGK1 (Hs99999906_m1); PKM2 (Hs00987255_m1); IDH3G (Hs00188065_m1); SDH (Hs01042482_m1); OGDH (Hs01081865); MDH2 (Hs00938918_m1); AMPK (Hs01562315_m1); TNF*α* (Hs00174128_m1); IL-1*β* (Hs00174097_m1); COL2A1 (Hs00264051_m1); and HIF1*α* (Hs00936368_m1). *β-Actin* was used as an endogenous control.

Quantification of gene expression levels of mRNA was conducted using a 7500 real-time PCR System (Applied Biosystems, Foster City, CA, USA). 1 *μ*L of RT product was subjected to real-time PCR in a 15 *μ*L total reaction mixture containing 7.5 *μ*L TaqMan Universal PCR Master Mix (Applied Biosystems, Foster City, CA, USA), 900 nM sense and antisense primers, and 50 nM probe. After a single step of 50°C for 2 min and initial activation at 95°C for 10 min, reaction mixtures were subjected to 40 amplification cycles (15 s at 95°C for denaturation and 1 min of annealing and extension at 60°C).

Using a sequence detection system, the threshold cycle (C_T_) was determined based on when the exponential amplification of the PCR products begins. After the PCR, dissociation curves were generated with one peak that indicated the specificity of the amplification. A threshold cycle (C_T_ value) was obtained from each amplification curve using SDS V 1.3 software provided by the manufacturer (Applied Biosystems, Foster City, CA, USA).

Relative mRNA expression was determined using the ddC_T_ method, as detailed by the manufacturer guidelines (Applied Biosystems, Foster City, CA, USA). A dC_T_ value was calculated by subtracting the C_T_ value for the housekeeping gene *β*-actin from the C_T_ value for each sample. A ddC_T_ value was then calculated by subtracting the dC_T_ value of the control (unstimulated cartilage) from the dC_T_ value of each treatment. Fold changes compared with the control were determined by raising 2 to the ddC_T_ power (2^−ddCT^). Three “no template” controls were consistently negative for each reaction. To avoid variation in efficiency between the experiments, all the samples of the same cartilage were simultaneously subjected to RT, and the cDNA samples were simultaneously amplified using real-time PCR. Cartilage obtained from each patient was cultured with or without DFO in triplicate for 48 h. Isolated RNA was assayed by RT-PCR in duplicate.

### 2.11. Statistical Analysis

Quantitative data were expressed as a mean ± standard deviation (SD). Statistica 10 for Windows (StatSoft Inc., USA) was used for all statistical analyses. A *p* value of 0.05 or less was considered statistically significant. A statistical comparison between the independent patient groups was performed using the Mann–Whitney *U* test. For the statistical comparison between the RA patient groups before and after treatment, the Wilcoxon matched pairs test was applied.

## 3. Results

### 3.1. Inhibition of Collagenase Activity by DFO

**Figure 1:**
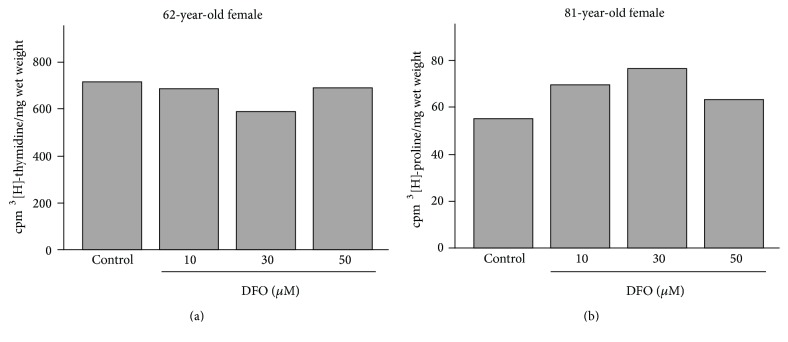
Representative analyses of ^3^[H]-thymidine incorporation (a) and ^3^[H]-proline uptake (b) into OA articular cartilage explants in the absence and presence of 10–50 *μ*M DFO.

Previously, DFO was used with chondrocytes in a variety of concentrations ranging from 10 nM to 500 *μ*M [29]. Therefore, we initially tested 1 to 50 *μ*M DFO concentrations for toxicity, examining the effects of the drug on cellular DNA and protein synthesis.  Figure 1 shows examples of these analyses performed on samples from 62-year-old and 81-year-old female OA patients. Repeat studies of other patients revealed essentially the same results; that is, DFO concentrations within this range were not toxic to articular cartilage chondrocytes in explant culture.

**Figure 2:**
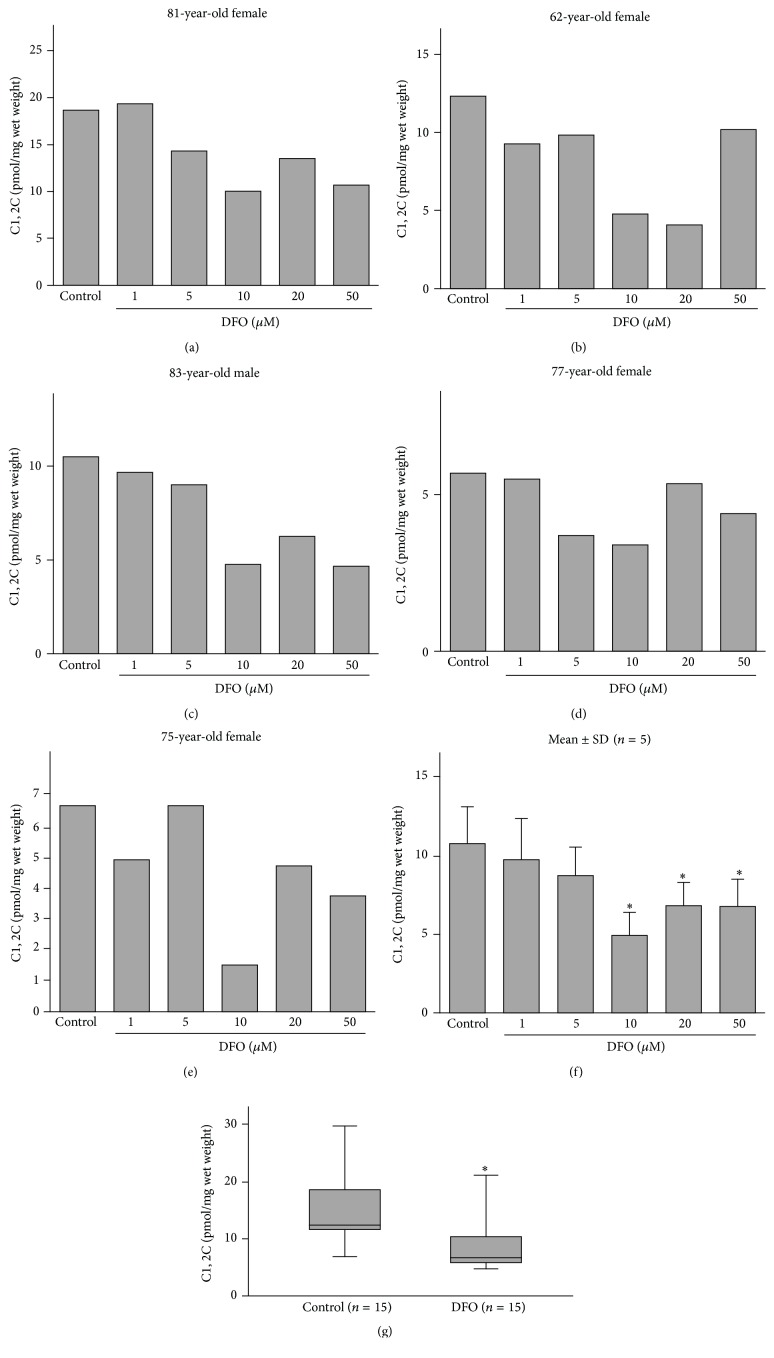
Deferoxamine (DFO) inhibition of type II collagen cleavage by collagenase in human osteoarthritic (OA) articular explants from patients whose ages and genders are indicated (a–e). The means ± SDs for all five patients are shown in (f) (Wilcoxon matched pairs test). Inhibition of type II collagen cleavage by collagenase in human OA articular explants by 10 *μ*M DFO. Cartilages from an additional 15 OA articular cartilage explants were analyzed after exposure to 10 *μ*M DFO. The means ± SDs are shown in (g) (Mann–Whitney *U* test). Significant differences compared to the control (*p* < 0.05) are indicated by asterisks (*∗*).

Our analyses of the effect of DFO dose on collagen cleavage revealed that although in one patient, a 5 *μ*M DFO downregulated collagenase activity as measured by the C1,2C method, inhibition of activity was observed with 10 *μ*M DFO and higher in other patients (Figures 2(a)–2(f)) and at 10 *μ*M dose in a larger cohort of 15 other patients (Figure 2(g)) in which a mean inhibition of 43.3% (range 27.6 to 61.5%) was noted.

### 3.2. Alterations of Gene Expression in OA Cartilage Explants by DFO

**Figure 3:**
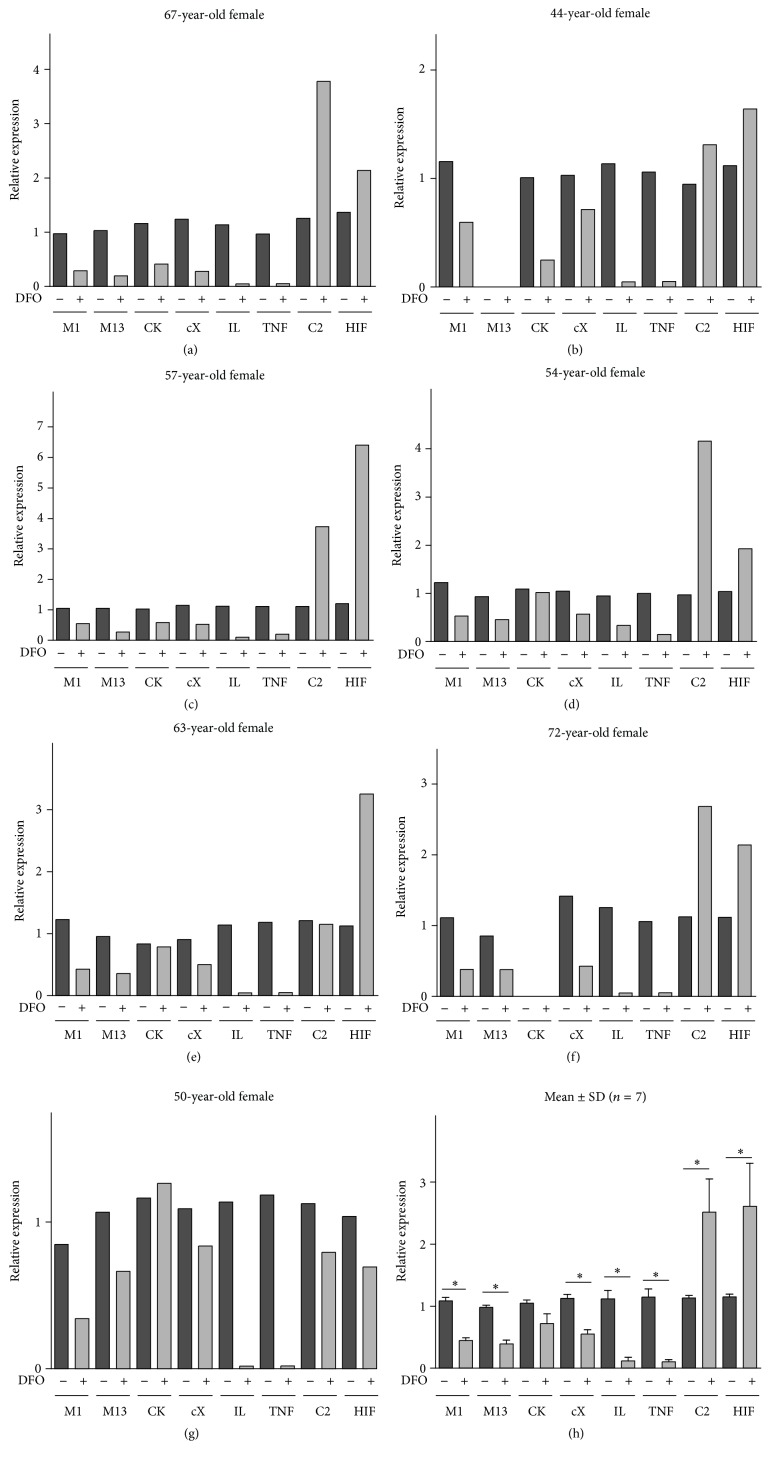
Effects of DFO on the expression of genes responsible for collagen cleavage, chondrocyte hypertrophy and inflammation, collagen synthesis, and hypoxia. Relative gene expression with reference to *β*-actin is compared with controls, as determined by real-time PCR analyses in osteoarthritic cartilage explant cultures in the presence or absence (control) of 10 *μ*M deferoxamine (DFO) at 48 hours. The means ± SDs for all seven patients (a–g) are shown in (h) (Wilcoxon matched pairs test). Asterisks (*∗*) indicate significant differences compared to the control (*p* < 0.05). The age and gender of each patient are indicated. M1, MMP-1; M13, MMP-13; CK, cathepsin K; cX, COL10A1; C2, COL2A1; HIF, HIF1*α*; IL, IL-1*β*; TNF, TNF *α*.

For gene expression studies, we examined cartilage explants from 67-, 44-, 63-, 72-, 50-, 57-, and 54-year-old patients, which exhibited significant inhibition of collagenase cleavage (55.4, 60.2, 54.0, 45.4, 43.7, 48.6, and 50.4%, resp.). Mankin grading of the examined OA cartilages has revealed values of 8.6, 10, 9,6, 10,3, 9, 7.3, and 7, respectively. Compared with the controls, when cultured in the presence of 10 *μ*M DFO, the OA explants from all 7 patients exhibited reduced expression of MMP-1 (Figures 3(a)–3(g), Table 1). The expression of the collagenase MMP-13 was downregulated in 6 of the 6 patients examined; the gene related to chondrocyte hypertrophy, COL10A1, in 7 of 7 subjects; and protease cathepsin K expression was reduced in 3 of 6 patients. In all patients examined, the gene expressions of the proinflammatory cytokines IL-1*β* and TNF*α* were downregulated by DFO. In contrast, expression of COL2A1 (5 of 7) and HIF1*α* (6 of 7) was upregulated in the same OA explants, although one patient exhibited decreased HIF1*α* and COL2A1 gene expression (Figures 3(a)–3(g), Table 1) and another patient exhibited no change in COL2A1 expression.

**Figure 4:**
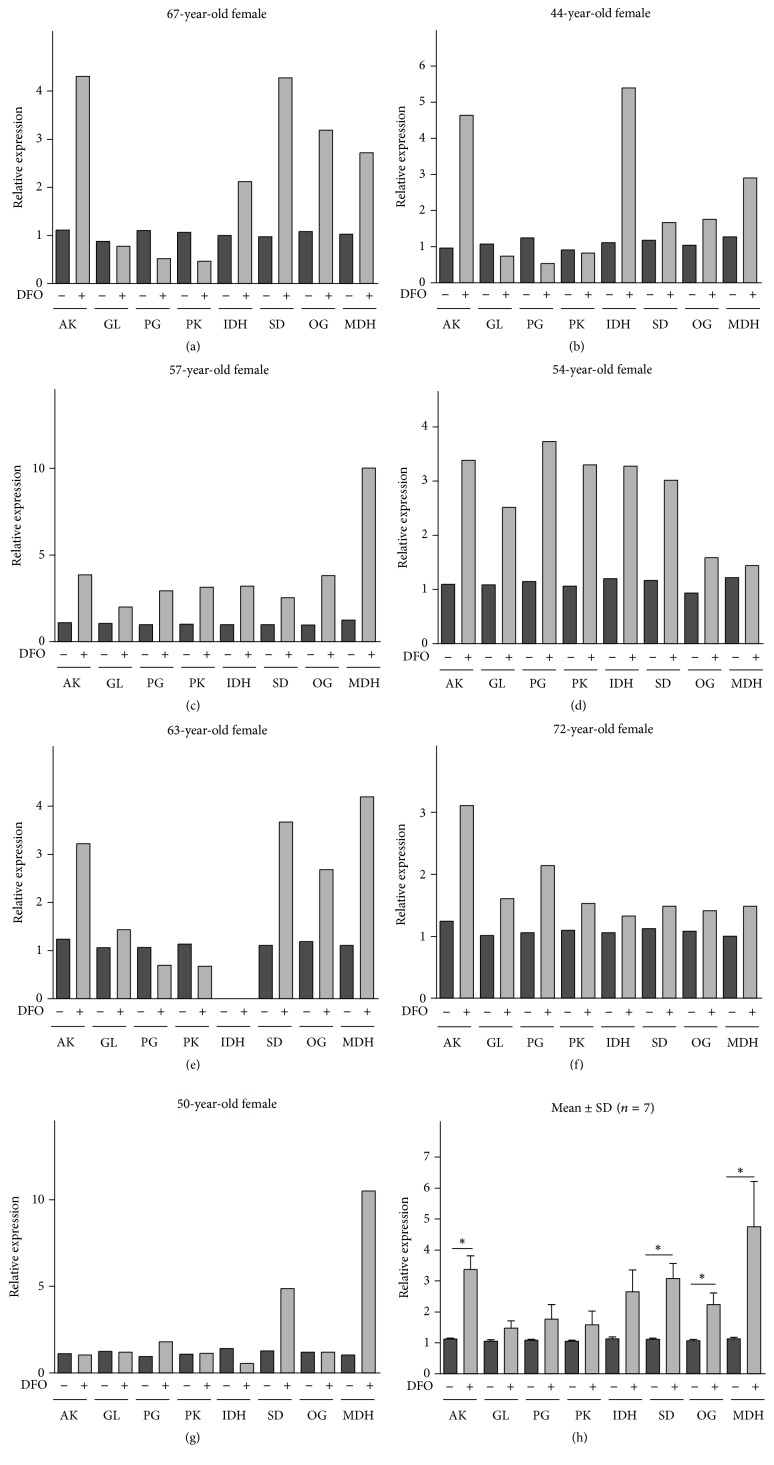
Effects of DFO on the expression of genes responsible for the TCA cycle, glycolysis, and AMPK. Relative gene expression with reference to *β*-actin is compared with controls, as determined by real-time PCR analyses in osteoarthritic cartilage explant cultures in the presence or absence (control) of 10 *μ*M deferoxamine (DFO) at 48 hours. The means ± SDs for all seven patients (a–g) are shown in (h) (Wilcoxon matched pairs test). Asterisks (*∗*) indicate significant differences compared to the control (*p* < 0.05). The age and gender of each patient are indicated. AK, AMPK; GL, Glut1; PG, PGK1; PK, PKM2; IDH, IDH3G; SD, SDH; OG, OGDH; MDH, MDH2.

DFO had no effect on the expression of the glucose transporter Glut1 in 3 of 7 patients (Figures 4(a)–4(g), Table 1). However, Glut1 was upregulated in 3 patients and slightly downregulated in another. Pyruvate kinase (PKM2), an ATP-producing gene involved in glycolysis, was downregulated in 2 of 7 patients, was upregulated in 3 patients, and showed no changes in other subjects (2 of 7). Another glycolysis gene associated with ATP generation, phosphoglycerate kinase 1 (PGK1), was upregulated in 4 of 7 OA patients and downregulated in 3 of 7 cases in the presence of DFO.

Regarding the genes involved in ATP generation in the TCA cycle, DFO upregulated malate dehydrogenase (MDH2) gene expression in 7 of 7 OA patients and succinate dehydrogenase (SDH) gene expression in all of the same patients. Isocitrate dehydrogenase (IDH3G) (5 of 6) and 2-oxo-glutarate dehydrogenase (OGDH) (6 of 7) were increased in most patients in response to DFO treatment (Figures 4(a)–4(g), Table 1). We also observed upregulation of AMPK gene expression in 6 of 7 patients (Figures 4(a)–4(g)).

The mean and SD values for these gene expression analyses are shown in Figures 3(h) and 4(h) where the interrelationships of these DFO effects can be more clearly observed. Overall, DFO significantly reduced the expression of collagenases MMP-1, MMP-13, cytokines IL-1*β* and TNF*α*, and COL10A1, while stimulating succinate dehydrogenase, 2-oxo-glutarate dehydrogenase, malate dehydrogenase, AMPK, COL2A1, and HIF1*α*. Gene expression of isocitrate dehydrogenase was also upregulated, but not significantly (*p* = 0.09) (Figure 4(h)).

### 3.3. AMPK Gene Expression in the PBMCs and Articular Cartilage of OA and Normal Patients

**Figure 5:**
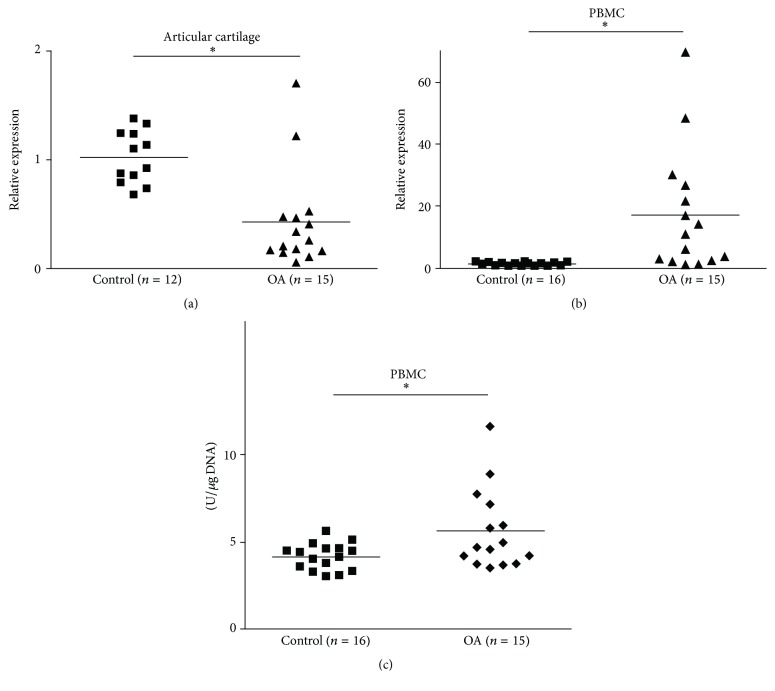
AMPK expression. Relative gene expression in (a) articular cartilages of healthy (*n* = 12) and OA patients (*n* = 15) and in (b) PBMCs of the same OA patients (*n* = 15) versus 16 healthy subjects, as determined by real-time PCR analyses with reference to *β*-actin. (c) pAMPK protein concentrations in PBMCs of the same OA patients (*n* = 15) versus 16 healthy subjects as determined by ELISA. Asterisks (*∗*) indicate significant differences from controls (Mann–Whitney* U* test).

A comparison of the expression of AMPK in OA articular cartilages (*n* = 15) with that in healthy subjects (*n* = 12) revealed significant downregulation from OA patients (Figure 5). In contrast, PBMCs of the same 15 OA patients exhibited a significant upregulation of AMPK gene expression compared to age- and gender-matched healthy controls (*n* = 16). Moreover, gene expression results for PBMCs were confirmed by examination of AMPK protein, which again revealed significantly higher AMPK protein concentrations in OA patients (*n* = 15) compared to healthy subjects (*n* = 16).

## 4. Discussion

It has been shown previously that downregulation of collagen cleavage in OA articular cartilage is associated with inhibition of inflammation, MMP expression, chondrocyte hypertrophy, and activation of AMPK [8, 25, 30]. Even* in vivo,* collagen cleavage in experimental OA can be reduced by inhibition of either MMP activity or chondrocyte hypertrophy [31, 32] favouring matrix repair. Other studies have reported that expression of ECM-related genes could be stimulated in a hypoxic environment via improved mitochondrial energy balance [12–14]. Therefore, we were interested in determining whether inhibition of collagen cleavage is related to increases in gene expression associated with collagen repair in an OA cartilage explant model and how this effect relates to energy metabolism.

Towards this end, we studied the effects of DFO, which upregulates HIF1*α* and glycolysis, improving aerobic respiration and tissue repair [18–23]. We conducted our studies under normoxic conditions because previous studies have shown that DFO is capable of upregulating HIF1*α* expression under conditions of normal oxygen tension [20, 21]. Besides, at low oxygen concentrations these effects might be due to a hypoxic response. At the same time, the observation that intracellular pAMPK concentrations are equal at 20% and 5% O2 in porcine articular chondrocytes [33] is important, as we wished to examine AMPK gene expression. Furthermore, we used a high glucose DMEM to provide glucose saturation to maintain high glycolytic activity, as OA chondrocytes have been shown to acquire ATP primarily via glycolysis [34].

Using this model, we show that DFO is capable of inhibiting excessive collagen cleavage in cultured human OA articular cartilage explants. This inhibition is accompanied by downregulation of collagenases MMP-1 and MMP-13; the cytokines IL-1*β* and TNF*α*; and COL10A1, a marker of chondrocyte hypertrophy. At the same time, we also observed significant increases in HIF1*α* and type II collagen (COL2A1) gene expression accompanied by upregulation of AMPK and TCA-related genes. The products of TCA-related genes may provide precursors for amino acid synthesis in the same OA articular cartilage explants. This may be important given that OA articular cartilage exhibits a significant inhibition of autophagy, a process involving the degradation and recycling of dysfunctional organelles and cellular breakdown products with the release of the resulting macromolecules back into the cytosol [35–37]. An increase in IDH3G in response to DFO is also important, as oxidative decarboxylation of isocitrate produces 2-oxoglutarate, a proline precursor, posttranslational hydroxylation of which is required for collagen synthesis [13]. Therefore, the observed increases in AMPK gene expression in 6 of 7 patients may be associated with elevated energy expenditure for protein synthesis [4], as evidenced by COL2A1 upregulation. Upregulation of AMPK expression may be pivotal for articular cartilage anabolic functions and restoration of a prehypertrophic chondrocyte phenotype as AMPK upregulation associated with OA articular cartilage repair was previously reported [9].

Although inhibition of collagen cleavage by DFO was associated with anticatabolic and anti-inflammatory effects in all of the OA patients studied, alterations in the expression of other genes varied. The general trend was observed in two patients aged 67 years old and 44 years old. In 72-year-old, 57-year-old, and 54-year-old patients, an increase in COL2A1 expression was associated with upregulation of HIF1*α* and its target genes Glut1 and PGK1 [38]. The latter genes are involved in glycolysis, which could also provide the amino acids required for protein synthesis via the pentose phosphate cycle, as observed in some types of cancer [39]. In contrast, in a 63-year-old patient, HIF1*α* upregulation was accompanied by no change in COL2A1 expression that resembled the chondrocyte response to o-phenanthroline treatment [15, 16]. This result may be associated with a shortage in proline precursors due to the disturbance in IDH3G expression, which was not detected in explants obtained from this patient. Finally, in a 50-year-old OA patient, downregulation of COL2A1, HIF1*α*, and IDH3G gene expression in response to DFO was associated with no change in AMPK expression, although TCA-related SDH and MDH2 genes were upregulated. These results show that metabolic responses in individual patients vary. Variability of patient responses to treatment tested in articular cartilage explants was also observed by us previously and might be due to differences in OA pathology [27, 40]. This further indicates the importance of personalized approach in individual patient treatment. However, the assessment of mean values in a group of patients might help to understand the general trend of the drug action. If upregulation of AMPK, HIF1*α*, and TCA-related gene expression is pivotal for promotion of collagen repair, then this is a testable hypothesis in future studies.

Although inflammation is believed to drive collagen cleavage in OA articular cartilage, we observed that IL-1*β* and TNF*α* downregulation was not always accompanied by decrease in expression of all the proteases in different patients. Such variability is not new. For example, in earlier OA studies of human articular cartilages, we observed differences between patients in the activities of different collagenases based on differential responses to a selective collagenase inhibitor [27]. In addition, previously we noted that joint destruction activity is more difficult to inhibit than inflammation [41].

Interpretation of these observations is complicated by the presence of chondrocytes of an altered phenotype, reflected in part by the proliferative changes characterized by the cell clusters seen in degraded articular cartilage, particularly in advanced OA [42], and by chondrocyte hypertrophy characterized by expression of COL10A1 [1]. Furthermore, these clusters can also contain mesenchymal stem cells, again with distinct metabolomes [43–45]. But since it is possible to reverse this hypertrophic phenotype of chondrocytes in association with inhibition of collagen degradation, as we have shown here, there is arguably potential to explore the shift of the phenotype to one where matrix anabolism is emphasized over matrix catabolism.

Unfortunately, we do not have access to obtain sufficient numbers of specimens to conduct studies on DFO effects on normal human articular cartilage. These studies should be definitely conducted in future.

As we studied cartilage specimens from OA patients, we are aware that the observed DFO effects occur in OA articular cartilage. What we are interested in is whether DFO can regulate molecular events that have previously been shown to be a feature of OA pathology. However, these effects might also happen at other pathologies involving articular cartilage degradation. Additional studies involving degrading articular cartilages from patients with other pathologies are required to examine this possibility.

Different types of behavior for AMPK in the peripheral blood and articular cartilage in OA patients compared to healthy controls are not a unique phenomenon as previously a similar type of behavior was observed in case of autophagy-related ULK1 gene expression in the end-stage OA patients [36]. Interestingly, both genes are associated with mTOR (mechanistic target of rapamycin) regulation: AMPK is an mTOR inhibitor [46] while an increase in autophagy is associated with mTOR inhibition upon cell growth cessation [47]. mTOR is a key regulator of cell growth and proliferation [48], which integrates contributions from amino acids, growth factors, and molecules involved in energy status of the cell [49] and is upregulated in both the articular cartilage and the peripheral blood of the end-stage OA patients [36]. Therefore, our observations suggest that OA articular cartilage maintained normal regulatory behavior while in the PBMCs this regulation might be disturbed as mTOR, AMPK, and ULK1 gene expressions are all upregulated compared to healthy subjects.

Our observation that reduced AMPK gene expression in OA articular cartilage is accompanied by its high expression in PBMCs in the same OA patients compared to healthy subjects suggests that whole-body energy availability and that in articular cartilage can differ in OA patients. This might induce an energy appeal reaction, a process that involves the redirection of energy-rich fuels from energy sources, such as articular cartilage in OA, as indicated by studies of the activated immune system [4, 50]. As catabolic pathways are responsible for energy provision [51], the excessive cartilage degradation seen in OA might represent an energy-rich compartment for further energy relocation to specific body sites, such as the immune system.

In summary, our studies demonstrate that DFO is capable of suppressing excessive collagenase-mediated type II collagen cleavage in OA cartilage and reversing phenotypic changes. The concomitant upregulation of proanabolic TCA-related gene expressions points to a potential for availability of energy generating substrates required for matrix repair by end-stage OA chondrocytes. This might normally be prevented by strong whole-body energy requirements indicated by elevated AMPK expression in PBMCs of OA patients. Further studies on the involvement of chondrocyte energy metabolic alterations in OA might provide new approaches for better disease control.

## Supplementary Material

Supplementary Figure. Diagrammatic representation of human femoral head. Black circles indicate the sites of articular cartilage withdrawal from normal knee of control patients.

## Figures and Tables

**Table 1:** Summary of gene expression in OA articular cartilage explants.

	Gene expression response in patients treated with DFO			
	Downregulation	Upregulation	No change	No expression
MMP-1	7	0	0	0
MMP-13	6	0	0	1
Cathepsin K	3	0	3	1
COL10A1	7	0	0	0
IL-1*β*	7	0	0	0
TNF*α*	7	0	0	0
Glut 1	1	3	3	0
PGK1	3	4	0	0
PKM2	2	3	2	0
IDH3G	1	5	0	1
SDH	0	7	0	0
OGDH	0	6	1	0
MDH2	0	7	0	0
AMPK	0	6	1	0
HIF1*α*	1	6	0	0
COL2A1	1	5	1	0

## Abbreviations

AMPK: AMP-activated protein kinase
ATP: Adenosine triphosphate
TCA: Citric acid cycle, tricarboxylic acid cycle, and Krebs cycle
cDNA: Complementary deoxyribonucleic acid
COL10A1: Type X collagen
COL2A1: Type II collagen
DFO: Deferoxamine
DMEM: Dulbecco's modified eagle medium A
ECM: Extracellular matrix
EDTA: Ethylenediaminetetraacetic acid
ELISA: Enzyme-Linked ImmunoSorbent Assay
GAPDH: Glyceraldehyde-3-phosphate dehydrogenase
HEPES: 4-(2-Hydroxyethyl)-1-piperazineethanesulfonic acid
IDH3G: Isocitrate dehydrogenase
IL-1*β*: Interleukin 1*β*
MDH: Malate dehydrogenase
MMP: Matrix metalloproteinase
mRNA: Messenger ribonucleic acid
OA: Osteoarthritis
OGDH: *α*-Oxoglutarate dehydrogenase
PCR: Polymerase chain reaction
PGK: Phosphoglycerate kinase
PKM: Pyruvate dehydrogenase
RNA: Ribonucleic acid
SDH: Succinate dehydrogenase
SDS: Sodium dodecyl sulfate
TNF*α*: Tumor necrosis factor *α*.

## References

[1] Poole A. R., Koopman W. (2005). Cartilage in health and disease. *Arthritis and Allied Conditions*.

[2] Tchetina E. V. (2011). Developmental mechanisms in articular cartilage degradation in osteoarthritis. *Arthritis*.

[3] Tchetina E. V., Squires G., Poole A. R. (2005). Increased type II collagen degradation and very early focal cartilage degeneration is associated with upregulation of chondrocyte differentiation related genes in early human articular cartilage lesions. *Journal of Rheumatology*.

[4] Spies C. M., Straub R. H., Buttgereit F. (2012). Energy metabolism and rheumatic diseases: from cell to organism. *Arthritis Research and Therapy*.

[5] Hardie D. G. (2008). AMPK: a key regulator of energy balance in the single cell and the whole organism. *International Journal of Obesity*.

[6] Tangeman L., Wyatt C. N., Brown T. L. (2012). Knockdown of AMP-activated protein kinase alpha 1 and alpha 2 catalytic subunits. *Journal of RNAi and Gene Silencing*.

[7] Lehninger L. A., Cox M. M., Nelson D. L. (2008). *Lehninger Principles of Biochemistry*.

[8] Terkeltaub R., Yang B., Lotz M., Liu-Bryan R. (2011). Chondrocyte AMP-activated protein kinase activity suppresses matrix degradation responses to proinflammatory cytokines interleukin-1*β* and tumor necrosis factor *α*. *Arthritis and Rheumatism*.

[9] Wang Y., Zhao X., Lotz M., Terkeltaub R., Liu-Bryan R. (2015). Mitochondrial biogenesis is impaired in osteoarthritis chondrocytes but reversible via peroxisome proliferator-activated receptor *γ* coactivator 1*α*. *Arthritis and Rheumatology*.

[10] Bohensky J., Leshinsky S., Srinivas V., Shapiro I. M. (2010). Chondrocyte autophagy is stimulated by HIF-1 dependent AMPK activation and mTOR suppression. *Pediatric Nephrology*.

[11] Fukuyama Y., Ohta K., Okoshi R., Suehara M., Kizaki H., Nakagawa K. (2007). Hypoxia induces expression and activation of AMPK in rat dental pulp cells. *Journal of Dental Research*.

[12] Pfander D., Cramer T., Schipani E., Johnson R. S. (2003). HIF-1*α* controls extracellular matrix synthesis by epiphyseal chondrocytes. *Journal of Cell Science*.

[13] Grimmer C., Balbus N., Lang U. (2006). Regulation of type II collagen synthesis during osteoarthritis by prolyl-4-hydroxylases: possible influence of low oxygen levels. *The American Journal of Pathology*.

[14] Markway B. D., Cho H., Johnstone B. (2013). Hypoxia promotes redifferentiation and suppresses markers of hypertrophy and degeneration in both healthy and osteoarthritic chondrocytes. *Arthritis Research and Therapy*.

[15] Georgi N., Landman E. B., Klein T. J., van Blitterswijk C. A., Karperien M. (2014). O-Phenanthroline as modulator of the hypoxic and catabolic response in cartilage tissue-engineering models. *Journal of Tissue Engeneering and Regenerative Medicine*.

[16] Xia M., Huang R., Sun Y. (2009). Identification of chemical compounds that induce HIF-1alpha activity. *Toxicological Sciences*.

[17] Butow R. A., Racker E. (1965). On the mechanism of respiratory control. *Journal of General Physiology*.

[18] Jiang L., Peng W. W., Li L. F. (2014). Effects of deferoxamine on the repair ability of dental pulp cells in vitro. *Journal of Endodontics*.

[19] Hou Z., Nie C., Si Z., Ma Y. (2013). Deferoxamine enhances neovascularization and accelerates wound healing in diabetic rats via the accumulation of hypoxia-inducible factor-1*α*. *Diabetes Research and Clinical Practice*.

[20] Wang G. L., Semenza G. L. (1993). Desferrioxamine induces erythropoietin gene expression and hypoxia-inducible factor 1 DNA-binding activity: implications for models of hypoxia signal transduction. *Blood*.

[21] Xiao H., Gu Z., Wang G., Zhao T. (2013). The possible mechanisms underlying the impairment of hif-1*α* pathway signaling in hyperglycemia and the beneficial effects of certain therapies. *International Journal of Medical Sciences*.

[22] Hervouet E., Cízková A., Demont J. (2008). HIF and reactive oxygen species regulate oxidative phosphorylation in cancer. *Carcinogenesis*.

[23] Fukuda R., Zhang H., Kim J.-W., Shimoda L., Dang C. V., Semenza G. (2007). HIF-1 regulates cytochrome oxidase subunits to optimize efficiency of respiration in hypoxic cells. *Cell*.

[24] Altman R., Asch E., Bloch D. (1986). Development of criteria for the classification and reporting of osteoarthritis: classification of osteoarthritis of the knee. *Arthritis and Rheumatism*.

[25] Tchetina E. V., Antoniou J., Tanzer M., Zukor D. J., Poole A. R. (2006). Transforming growth factor-*β*2 suppresses collagen cleavage in cultured human osteoarthritic cartilage, reduces expression of genes associated with chondrocyte hypertrophy and degradation, and increases prostaglandin E2 production. *American Journal of Pathology*.

[26] Yasuda T., Tchetina E., Ohsawa K. (2006). Peptides of type II collagen can induce the cleavage of type II collagen and aggrecan in articular cartilage. *Matrix Biology*.

[27] Dahlberg L., Billinghurst R. C., Manner P. (2000). Selective enhancement of collagenase-mediated cleavage of resident type II collagen in cultured osteoarthritic cartilage and arrest with a synthetic inhibitor that spares collagenase 1 (Matrix metalloproteinase 1). *Arthritis and Rheumatism*.

[28] Son B. K., Roberts R. L., Ank B. J., Stiehm E. R. (1996). Effects of anticoagulant, serum, and temperature on the natural killer activity of human peripheral blood mononuclear cells stored overnight. *Clinical and Diagnostic Laboratory Immunology*.

[29] Hatori M., Sparkman J., Teixeira C. C. (1995). Effects of deferoximine on chondrocyte alkaline phosphatase activity: proxidant role of deferoximine in thalassemia. *Calcified Tissue International*.

[30] Tchetina E. V., Di Battista J. A., Zukor D. L. (2007). Prostaglandin PGE_2_ at very low concentrations suppresses collagen cleavage in cultured human osteoarthritic articular cartilage: this involves a decrease in expression of proinflammatory genes, collagenases and *COL10A1*, a gene linked to chondrocyte hypertrophy. *Arthritis Research and Therapy*.

[31] Little C. B., Fosang A. J. (2010). Is cartilage matrix breakdown an appropriate therapeutic target in osteoarthritis-insights from studies of aggrecan and collagen proteolysis?. *Current Drug Targets*.

[32] Bondeson J. (2011). Are we moving in the right direction with osteoarthritis drug discovery?. *Expert Opinion on Therapeutic Targets*.

[33] Fermor B., Gurumurthy A., Diekman B. O. (2010). Hypoxia, RONS and energy metabolism in articular cartilage. *Osteoarthritis and Cartilage*.

[34] Pfander D., Cramer T., Swoboda B. (2005). Hypoxia and HIF-1*α* in osteoarthritis. *International Orthopaedics*.

[35] Klionsky D. J., Abdalla F. C., Abeliovich H. A. (2016). Guidelines for the use and interpretation of assays for monitoring autophagy. *Autophagy*.

[36] Tchetina E. V., Poole A. R., Zaitseva E. M. (2013). Differences in *mTOR* (mammalian target of rapamycin) gene expression in the peripheral blood and articular cartilages of osteoarthritic patients and disease activity. *Arthritis*.

[37] Caramés B., Hasegawa A., Taniguchi N., Miyaki S., Blanco F. J., Lotz M. (2012). Autophagy activation by rapamycin reduces severity of experimental osteoarthritis. *Annals of the Rheumatic Diseases*.

[38] Makanji Y., Tagler D., Pahnke J., Shea L. D., Woodruff T. K. (2014). Hypoxia-mediated carbohydrate metabolism and transport promote early-stage murine follicle growth and survival. *American Journal of Physiology-Endocrinology and Metabolism*.

[39] Rahman M., Hasan M. R. (2015). Cancer metabolism and drug resistance. *Metabolites*.

[40] Kobayashi M., Squires G. R., Mousa A. (2005). Role of interleukin-1 and tumor necrosis factor *α* in matrix degradation of human osteoarthritic cartilage. *Arthritis and Rheumatism*.

[41] Tchetina E. V., Demidova N. V., Karateev D. E., Nasonov E. L. (2013). Rheumatoid factor positivity is associated with increased joint destruction and upregulation of matrix *metalloproteinase* 9 and *cathepsin* K gene expression in the peripheral blood in rheumatoid arthritic patients treated with methotrexate. *International Journal of Rheumatology*.

[42] Mitchell N., Lee E. R., Shepard N. (1992). The clones of osteoarthritic cartilage. *Journal of Bone and Joint Surgery B*.

[43] Pers Y.-M., Ruiz M., Noël D., Jorgensen C. (2015). Mesenchymal stem cells for the management of inflammation in osteoarthritis: state of the art and perspectives. *Osteoarthritis and Cartilage*.

[44] Zhong L., Huang X., Karperien M., Post J. N. (2015). The regulatory role of signaling crosstalk in hypertrophy of MSCs and human articular chondrocytes. *International Journal of Molecular Sciences*.

[45] Tang X., Fan L., Pei M., Zeng L., Ge Z. (2015). Evolving concepts of chondrogenic differentiation: history, state-of-the-art and future perspectives. *European Cells and Materials*.

[46] Gwinn D. M., Shackelford D. B., Egan D. F. (2008). AMPK phosphorylation of raptor mediates a metabolic checkpoint. *Molecular Cell*.

[47] Zhang M., Zhang J., Lu L. (2013). Enhancement of chondrocyte autophagy is an early response in the degenerative cartilage of the temporomandibular joint to biomechanical dental stimulation. *Apoptosis*.

[48] Hay N., Sonenberg N. (2004). Upstream and downstream of mTOR. *Genes and Development*.

[49] Marshall S. (2006). Role of insulin, adipocyte hormones, and nutrient-sensing pathways in regulating fuel metabolism and energy homeostasis: a nutritional perspective of diabetes, obesity, and cancer. *Science's STKE*.

[50] Straub R. H., Cutolo M., Buttgereit F., Pongratz G. (2010). Energy regulation and neuroendocrine-immune control in chronic inflammatory diseases. *Journal of Internal Medicine*.

[51] Straub R. H. (2011). Concepts of evolutionary medicine and energy regulation contribute to the etiology of systemic chronic inflammatory diseases. *Brain, Behavior, and Immunity*.

