# Supplementary material for: Deferoxamine Suppresses Collagen Cleavage and Protease, Cytokine, and COL10A1 Expression and Upregulates AMPK and Krebs Cycle Genes in Human Osteoarthritic Cartilage
Source: Int J Rheumatol. 2016 Nov 30;2016:6432867. doi: 10.1155/2016/6432867 (PMC5155111; doi:10.1155/2016/6432867)
Supplement: Supplementary file 1 — Supplementary Figure. Diagrammatic representation of human femoral head. Black circles indicate the sites of articular cartilage withdrawal from normal knee of control patients. [file 6432867.f1.pdf]

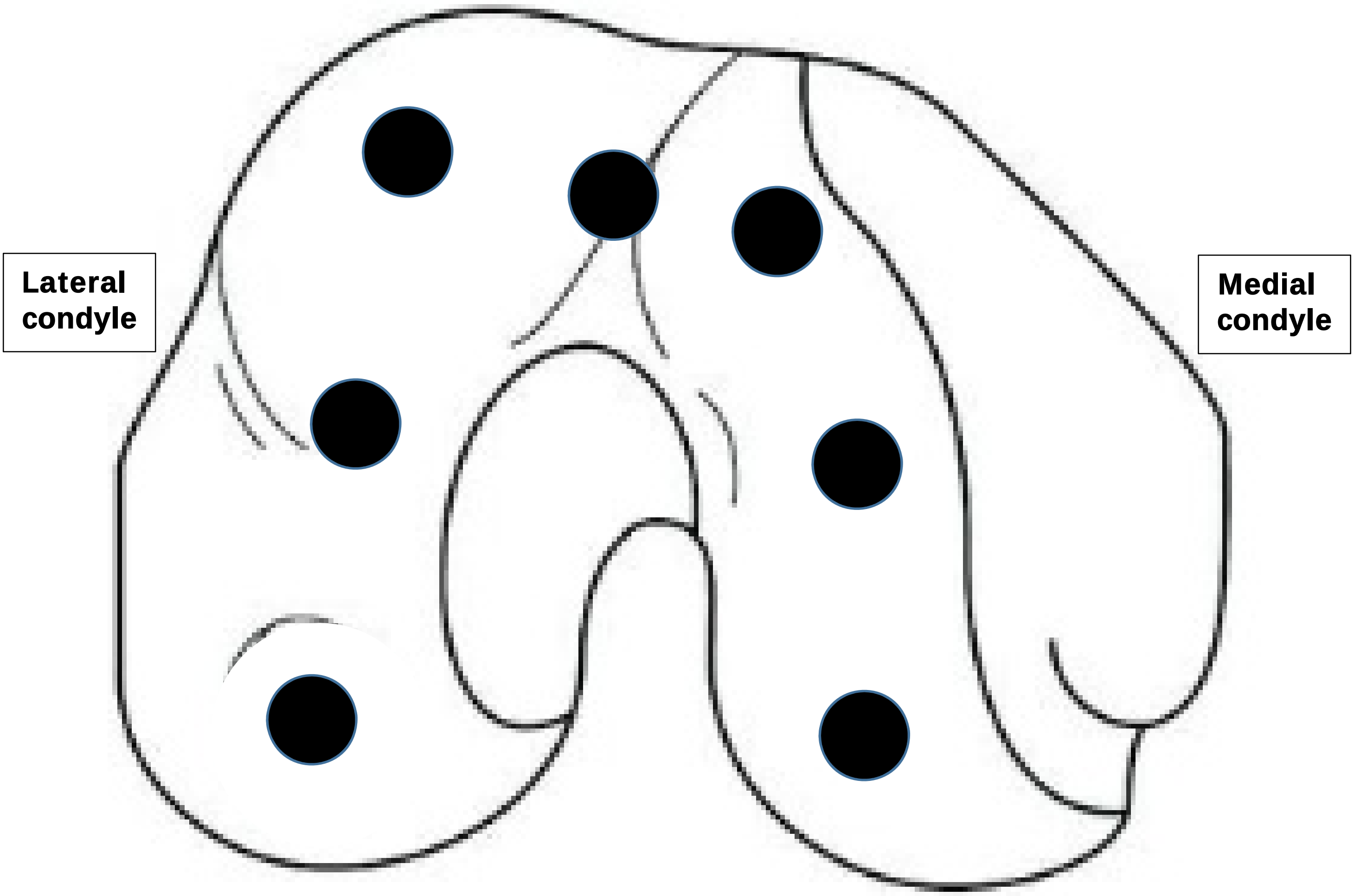

Supplementary Figure. Diagrammatic representation of human femoral head. Black circles indicate the sites of articular cartilage withdrawal from normal knee of control patients.
